# Comprehensive Analysis of Interactions between the Src-Associated Protein in Mitosis of 68 kDa and the Human Src-Homology 3 Proteome

**DOI:** 10.1371/journal.pone.0038540

**Published:** 2012-06-20

**Authors:** Benedikt Asbach, Christine Ludwig, Kalle Saksela, Ralf Wagner

**Affiliations:** 1 Institute of Medical Microbiology and Hygiene, University of Regensburg, Regensburg, Germany; 2 Department of Virology, Haartman Institute, University of Helsinki, Helsinki, Finland; Technical University of Braunschweig, Germany

## Abstract

The protein Sam68 is involved in many cellular processes such as cell-cycle regulation, RNA metabolism, or signal transduction. Sam68 comprises a central RNA-binding domain flanked by unstructured tails containing docking sites for signalling proteins including seven proline-rich sequences (denoted P0 to P6) as potential SH3-domain binding motifs. To comprehensively assess Sam68-SH3-interactions, we applied a phage-display screening of a library containing all approx. 300 human SH3 domains. Thereby we identified five new (from intersectin 2, the osteoclast stimulating factor OSF, nephrocystin, sorting nexin 9, and CIN85) and seven already known high-confidence Sam68-ligands (mainly from the Src-kinase family), as well as several lower-affinity binders. Interaction of the high-affinity Sam68-binders was confirmed in independent assays *in vitro* (phage-ELISA, GST-pull-down) and *in vivo* (FACS-based FRET-analysis with CFP- and YFP-tagged proteins). Fine-mapping analyses with peptides established P0, P3, P4, and P5 as exclusive docking-sites for SH3 domains, which showed varying preferences for these motifs. Mutational analyses identified individual residues within the proline-rich motifs being crucial for the interactions. Based on these data, we generated a Sam68-mutant incapable of interacting with SH3 domains any more, as subsequently demonstrated by FRET-analyses. In conclusion, we present a thorough characterization of Sam68’s interplay with the SH3 proteome. The observed interaction between Sam68 and OSF complements the known Sam68-Src and OSF-Src interactions. Thus, we propose, that Sam68 functions as a classical scaffold protein in this context, assembling components of an osteoclast-specific signalling pathway.

## Introduction

Many aspects of cell biology are controlled by regulatory mechanisms that form highly intertwined and complex signal transduction networks. Signal relay often occurs via protein-protein interactions that frequently employ conserved modular domains like the famous src-homology domains SH2 and SH3, that likewise recognize short conserved motifs, namely phosphotyrosines and proline-rich sequences, respectively [1]. SH3 domains consist of approx. 60 amino acids and usually exhibit a conserved fold with a core made up of five anti-parallel beta-strands. The surface comprises two hydrophobic pockets that generally recognize the common PxxP-ligand-motif (see below), and a specificity pocket for differential recognition of the respective target. Two variable loops, the so-called RT- and n-src-loops, mainly contribute to the specificity [2]. The central PxxP-motif in the target sequence forms a left-handed poly-proline type II helix with a hydrophobic face fitting into the SH3 domain’s hydrophobic pockets. Often, the PxxP is flanked by a basic amino acid that specifically interacts with an acidic RT-loop residue, thus defining the orientation of SH3-ligand binding. Depending on the location of this basic residue, ligand sequences are classified as class I (+xxPxxP consensus) or class II (PxxPx+) motifs [3]. In some cases, the basic residue is missing, and SH3 binding may even be idependent of a core PxxP [4]. SH3-PxxP interactions are usually described as quite weak with Kd-values in the micromolar range [2], however there are exceptions to this theme, like e.g. binding of the Hck-SH3-domain to the HI-viral Nef protein with a Kd of 250 nM [5].

A protein comprising an exceptionally large number of PxxP motifs is Sam68 (»src-associated in mitosis, 68 kDa«, systematically designated as KHDRBS1 for »KH domain containing, RNA binding, signal transduction associated 1«). It is involved in multiple cellular processes (reviewed in [6]), like signal transduction, cell cycle regulation, and RNA metabolism. Devoid of an enzymatic activity, Sam68 functions as an adaptor molecule mediating numerous protein- and RNA-interactions.

Sam68 consists of 443 amino acids corresponding to a mass of 48.2 kDa, though exhibiting an apparent size of approx. 68 kDa in SDS-PAGE analyses. The protein contains a central KH domain being responsible for the RNA-binding activity [7], which is embedded between two conserved regions termed NK and CK (for N-, or C-terminal of KH, respectively). Altogether they form the so-called GSG (GRP33, Sam68, GLD-1 domain) domain [8], that also mediates oligomerization [9] (most likely dimerization according to [10]). The C-terminal part of Sam68 contains a tyrosine-rich region, serving as docking site for SH2 domains after tyrosine-phosphorylation [11], as well as a nuclear localization sequence at the far end [12]. Sam68 is therefore supposed to reside mostly, however not exclusively, in the nucleus, depending on the cell cycle stage and protein modifications [13]. Furthermore, RG-rich sequences can be found in the N- and C-terminal part, that are involved in RNA binding. Arginine methylation here leads to a decrease, while lysine-acetylation of Sam68 leads to an increase in RNA binding activity [14], [15]. As already mentioned, Sam68 contains seven PxxP motifs (designated P0 to P6, see. Table S1) that serve as docking sites for various SH3 domains (see. Table S2).

The complexity of the diverse protein- and RNA-interactions, as well as the post-translational modification and subcellular localization patterns, is mirrored in the multi-faceted physiological roles of Sam68. It is implicated in several signal transduction processes, like insulin-, leptin-, EGF- or T-cell-receptor signalling, whose activations cause tyrosine-phosphorylation of Sam68 [16]–[19]. Furthermore Sam68 is involved in cell cycle control, concerning mitosis as well as meiosis. The role in the former is discussed somewhat controversially with reports of Sam68 being involved in cell-cycle progression or retardation [20]–[24]. Accordingly, Sam68 has been implicated in tumorigenesis, for example being upregulated in prostate carcinoma cells [25]. The role during meiosis has been thoroughly studied in the context of spermatogenesis (reviewed in [26]), which is disturbed in male Sam68^−/−^ knock-out mice causing infertility [27]. Alongside, these mice only display mild phenotypes, including a beneficial form of osteopetrosis and minor defects in motor coordination [28], [29]. Moreover, Sam68 plays an important role in RNA metabolism, especially in conjunction with alternative splicing. For instance, extracellular signals can activate ERK to phosphorylate Sam68, provoking inclusion of the v5 exon in a CD44 reporter system [30]. Finally, Sam68 is involved in the nuclear export of lentiviral RNAs.

To comprehensively analyse the SH3 domain interaction properties of Sam68, we performed a phage-display-based screening approach, followed by a thorough characterization of the identified binders. Besides confirming known SH3 domains as Sam68-binders, several new ones are described. Detailed analyses of Sam68-mutants reveal the individual PxxP motifs involved in the different SH3 interactions. Based on the fine-mapping of residues crucial for binding, we designed Sam68-mutants incapable of interacting with SH3 domains any more. The observed breadth of SH3 interactions is indicative of a model considering Sam68 as a classical scaffold protein.

## Methods

### Construction of Plasmids

The *sam68* gene was amplified via PCR from cDNA obtained from HEK293T-cells and inserted into the prokaryotic expression vector pQE-30 (Qiagen) via BamHI/BclI and SphI for recombinant production of N-terminally His-tagged Sam68; into pGEX-KG (GE Healthcare) via BamHI/BclI and EcoRI for recombinant production of N-terminally GST-tagged Sam68; into pECFP, or pEYFP (Takara) via EcoRI and KpnI for eukaryotic expression of N-terminally CFP/YFP-tagged Sam68. Sam68-mutants were generated by fusion-PCR using oligonucleotides with the desired mutations and reintroduction into the respective vector. Phagemids based on pJH containing the human *sh3* genes [31] were synthesized by Geneart AG. The respective *sh3* genes were amplified via PCR from these vectors and inserted into pGEX-KG via BamHI and EcoRI for recombinant production of N-terminally GST-tagged SH3 domains. Likewise, oligonucleotides coding for the Sam68-Px-peptides were annealed and directly inserted into pGEX-KG. Furthermore, selected *sh3* genes were introduced via BglII/BamHI and EcoRI into pEYFP for eukaryotic expression of N-terminally tagged YFP-SH3-domains.

### Protein Production

The production of N-terminally His-tagged Sam68 and the purification were carried out according to the QIAexpressionist handbook (Qiagen). Briefly, the cleared lysates from *E. coli* M15[pREP4] expression cultures were incubated with Ni-NTA-agarose (Qiagen); after thorough washing, bound protein was eluted from the beads with an excess of imidazole and dialysed against PBS for further use.

Production and purification of GST-tagged Sam68, SH3 domains or Px-peptides was carried out according to the GST-protein purification manual (GE Helthcare). Briefly, the cleared lysates from *E. coli* strain BL21 expression cultures were loaded onto a glutathion-sepharose column. After thorough washing bound proteins were eluted with an excess of glutathione and dialysed against PBS for further use. Protein concentrations were determined with the Bio-Rad protein assay (Bio-Rad).

### Phage Display

The bio-panning procedure to select SH3-domains binding to Sam68 was carried out essentially as described [31] with minor modifications: 10 µg His-Sam68 or GST-Sam68 were immobilized on 10^8^ magnetic M-270 epoxy beads (Dynal, Invitrogen) according to the manufacturer’s instructions. After blocking with a 5 % BSA in PBS solution, 200 µl of the SH3 phage library (Geneart, titer 6×10^10^ cfu/ml) diluted 1∶2 in blocking solution were added and shaken for 1 h. After 10 rounds of thorough washing with PBS +0.05 % Tween-20, retained phages were eluted by addition of 100 µl 200 mM Glycin, pH 2.2, for 10 min. The elution was neutralized by addition of 30 µl 1 M Tris, pH 9, and used to infect freshly grown *E. coli* TG1 cells (logarithmic phase, OD_600_ = 0.4 on a Bio-Rad SmartSpecPlus photometer). Bacteria were plated on SOBAG_Amp_ plates and incubated at 30°C over night. For identification of the corresponding *sh3* genes, phagemids were isolated according to standard procedures and analysed by sequencing. Phage supernatants derived from individual clones were produced by growing the bacteria in 2×YT_Amp,Glucose_ until OD  = 0.4 at 37°C and 220 rpm, followed by super-infection with 10^9^ cfu/ml M13KO7 helper phages under shaking for 30 min, exchanging the medium to 2×YT_Amp,Kana_, and incubating over night at 30°C and 220 rpm. Eventually the supernatant was cleared by filtration through a 0.45 µm filter and the phage titer determined by measuring infectious units in TG1 cells.

### Phage-ELISA

To characterize the binding of SH3-phages to recombinant proteins, phage-ELISA analyses were performed. First, 1 µg of recombinant protein per well was immobilized on 96-well MaxiSorp plates (Nunc) over night. After washing thrice with PBS/T (PBS with 0.1 % Tween-20) and blocking with 5 % BSA in PBS, dilution-series of the respective phage-supernatants in 2×YT_Amp,Kana_ were added and incubated for 1 h. After washing 10 times with PBS/T, an HRP-coupled anti-M13-antibody (GE Healthcare, 27-9421-01) diluted 1 : 5000 in blocking solution was added for 1 h. After washing again 10 times, TMB substrate solution was added, the reaction finally stopped by addition of 0.5 M H_2_SO_4_, and the result read out by measuring OD_450_.

### Cell Culture and Transfections

Human embryonal kidney 293T cells (ATCC-# CRL-11268) were cultivated according to standard procedures. Transfections were performed using the calcium-phosphate precipitation technique [32]. Cells were analyzed 48 h after transfection. Cell lysates from the human T-cell line MT-4 (NIH AIDS Research and Reference Reagent Program, Nr. 120) [33] for pull-down assays were obtained, after washing cells in ice cold PBS twice, by incubation with lysis-buffer (50 mM Tris, pH 8.0, 150 mM NaCl, 0.1 % SDS, 1 % Nonidet P-40, 0.5 % sodiumdesoxycholate) supplemented with protease inhibitors (Complete Mini, Roche) for 15 min with repeated vortexing. Finally, lysates were cleared by centrifugation.

### Pull-down Assay and Western Blots

10 µg of the recombinantly produced GST-SH3-domains each were immobilized on 10^8^ M-270 epoxy beads (see above). After blocking, 500 µl cell lysate (adjusted to 5 µg/ml total protein in lysis buffer) were added, and the beads shaken at 4°C over night. After washing thrice with PBS, retained proteins were eluted by addition of 25 µl SDS-PAGE sample buffer and incubation at 95°C for 5 min. The elutions were directly subjected to western blot analysis for detection of Sam68. Semi-dry western blots were performed according to standard protocols. For detection of Sam68, anti-Sam68 C20 antibody (Santa Cruz, sc-333, 1:5000 in TBS) was used in combination with an anti-rabbit-HRP secondary antibody (Pierce, 31460, 1∶5000 in TBS), followed by enhanced chemiluminescence detection using the Chemilux Pro device (Intas).

### FRET Analysis

A flow-cytometry-based FRET procedure to detect protein interactions in living cells was adapted from [34]. Briefly, cells were co-transfected with corresponding pairs of YFP- (yellow fluorescent protein) and CFP- (cyan fluorescent protein) tagged proteins, or a YFP-CFP-fusion protein as positive control, and harvested by trypsinization and gathering in FACS-buffer 48 h later for analysis with a FACSCanto II device (BD Biosciences). Excitation of CFP occurred at 405 nm, whereupon emission was detected in a BP450/50 filter (CFP only) and simultaneously in a BP585/42 filter (CFP and YFP). If FRET occurs, CFP emission decreases, while simultaneously YFP-emission increases. This can be visualized by a shift of the population distribution in a BP450/50 vs. BP585/42 fluorescence intensity plot, and be quantified by applying suitable gates based on negative control cells which have been transfected with CFP+YFP (for Sam68 interactions), or CFP-Sam68+YFP (for Sam68-PxxP-mutant interactions), so that the fraction of cells in R3 is below 0.1 %. In parallel, YFP is excited independently at 488 nm with detection in a BP530/30 filter as control.

## Results

### Phage-display Based Screening for Sam68-binding SH3 Domains

Several proteins that bind to Sam68 via an SH3 domain have formerly been described in the literature (see. Table S2). However, these studies focussed on single or few Sam68-binding proteins, while a systematic and comprehensive analysis of Sam68’s SH3-interactions is still missing. Therefore, we applied a phage-display-based screening of Sam68 against a library containing the near-complete human SH3 proteome according to a procedure by Kärkkäinen[/LOOSER] *et al.* [31]. Any of the 296 SH3 domains in this library is produced as a fusion with the major coat protein pVIII for display on the surface of bacteriophage M13. For the bio-panning procedure, briefly, recombinant Sam68 produced in *E. coli* and purified via an N-terminal His-tag or GST-tag was immobilized on magnetic epoxy-activated beads and incubated with the library (6×10^9^ cfu M13-pVIII-SH3 phages). After rigorous washing, retained phages were eluted from the beads by lowering pH, and subsequently used to infect fresh *E. coli* TG1 cells. The titers of the phage-elutions were 2.3×10^7^ cfu/ml for His-Sam68 and 2.1×10^7^ cfu/ml for GST-Sam68, as opposed to 2.2×10^5^ cfu/ml for the control-protein GST, which does not contain SH3 target PxxP motifs, thus hinting at specific enrichment of Sam68-binders. Phagemids from 162 of the obtained colonies were isolated and the identity of the SH3 domains determined by sequencing of the corresponding *sh3* genes. Candidates were considered as high confidence binders, if they were identified at least four times among these sequences, as the stochastic probability to obtain this frequency by chance from an evenly distributed library is less than 1% (binomial distribution with p = 1/296 and n = 162). The identities and frequencies of occurrence for these candidates are listed in Table 1, full results are shown in. Table S3. None of the domains was found among 20 sequences analysed from the GST-control, thus ruling out non-specific enrichment due to methodological constraints.

**Table 1:** Sam68-binding SH3 domains as identified by bio-panning of recombinant Sam68 against the human SH3-proteome phage-display library.

Nr.^a^	SH3 Domain from	Acc.-Nr.	Frequency	Known^b^
132	Lyn	P07948	16	+
292	Yes	P07947	13	+
95	Fyn	P06241	11	+
182	p85α	P27986	11	+
122	Intersectin 2 #3	O95062	8	
252	Src	P12931	7	+
106	Hck	P08631	7	+
162	Nephrocystin	O14837	6	
249	Sorting nexin 9	Q9Y5X1	5	
155	Nck1 #2	Nck1 #2	5	+
170	Osteoclast stimulatingfactor 1	Q92882	5	
37	CIN85 #1	Q9NYR0	4	
	4 SH3 domains^c^		3 each	1
	4 SH3 domains^c^		2 each	
	40 SH3 domains^c^		1 each	7

a Numbers refer to supplementary table from [31].

b SH3 domains already reported as Sam68-binders, compare suppl. Table S2.

c For a complete listing see suppl. Table S3.

In total, 12 different high confidence SH3 domains were identified. The large number of different binding partners is consistent with the observed breadth deduced from the literature. As highlighted in Table 1, seven of the top twelve identified SH3 domains have already been described as Sam68-binders. This concurrence confirms the fidelity of the applied bio-panning procedure. However, due to the limited number of clones analysed, more Sam68-binders – especially those with lower affinity – may remain undefined, like for instance some of the already described Sam68-binders (cf.Table S2). For a complete picture, unbiased separate analyses of all SH3 domains would be necessary, e.g. by performing microarray analyses as described in principle in [35], [36]. Five of the top-binders are to our knowledge described for the first time: SH3 domain #3 from Intersectin 2; Nephrocystin; Sorting nexin 9 (SN9); the osteoclast-stimulating factor 1; and SH3 domain #1 from Cbl-interacting 85 kDa protein (CIN85).

### Characterization of High-affinity Sam68-binders in vitro

For a detailed characterization of Sam68-binders we focussed the further experiments on a panel of ten SH3 domains, containing the top 7 binders, Nck1#2, the osteoclast stimulating factor 1 (OSF), as well as the SH3 domain from RasGAP as a negative control, since it does not interact with Sam68 according to [35], [37], nor was it obtained in the bio-panning. We chose Nck1 SH3 domain #2 (out of three) for further characterization, since Lawe *et al*. have described an interaction only for SH3 domain #1 [38], aiming to double-check this contradiction. OSF is a highly interesting candidate due to the link between Sam68 and bone metabolism (see discussion).

**Figure 1:**
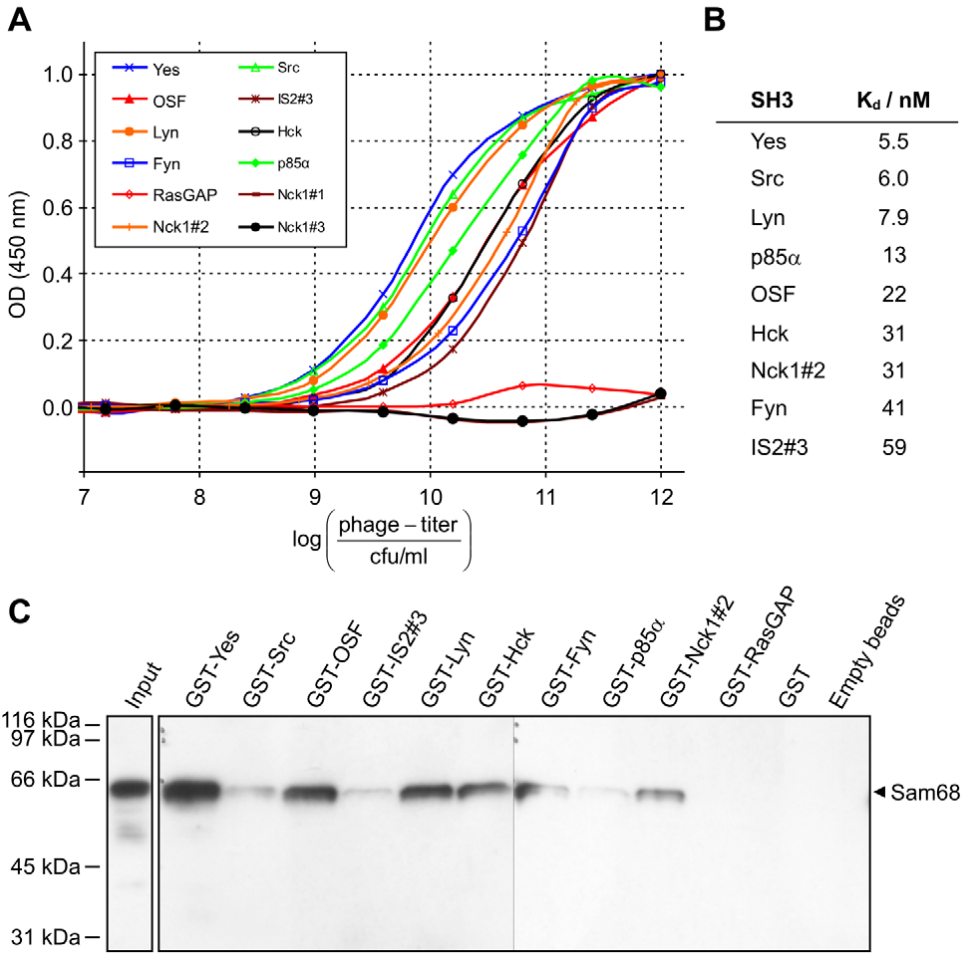
Analysis of Sam68-binding SH3 domains *in vitro*. (A) Phage-ELISA analysis: Recombinant Sam68 was coated in 96-well-plates and incubated with dilution series of individual SH3-phage supernatants. The amounts of bound phages were measured using a horseradish peroxidase-conjugated anti-M13-specific antibody with TMB-substrate detection at 450 nm (mean of three independent experiments, normalized to maximum OD-values). (B) Apparent K_d_-values for Sam68-SH3-phage-interactions were derived from the corresponding Scatchard-Plots (suppl. Fig. S2) to the data from (A), accounting for correction of SH3 domain concentrations. (C) Pull-down assay: Purified GST-SH3 domains were immobilized on magnetic beads and incubated with an MT-4 cell lysate. Bound proteins were eluted and Sam68 was detected by western blot analysis.

First, we aimed at quantifying the binding affinities of the SH3-displaying phages to Sam68. To this end, we established a phage-ELISA procedure to separately analyse phage supernatants of the ten individual SH3-phages. In brief, His-Sam68 was coated to a 96-well plate, blocked and incubated with a dilution series of phage supernatants. Finally, bound phages were detected using an anti-M13-specific HRP-coupled antibody. As depicted in Fig. 1A, binding curves were obtained that are typical for a simple ligand-receptor relationship as is expected for an SH3-domain-PxxP-interaction showing no allosteric effects. Kd-values can not be deduced from these curves in the first instance, because the concentration of SH3 domains in the supernatants is unknown due to each phage particle presenting many copies on its surface. To approximate Kd values, we performed an analogous analysis for the interaction of the Hck-SH3-phage with the HI-viral Nef-protein, since the affinity of the Nef-Hck-SH3 interaction is well characterized with a reported Kd-value of 250 nM [5]. Taking this value into account, evaluation of the binding curve for this interaction (see Figure S1) reveals that the phages carry approximately 420 SH3 domains per particle on average. Assuming that this value is true for all SH3 domains, which seems to be justified considering very similar sizes and common protein-structures, apparent Kd-values for the Sam68-SH3 interactions can be deduced from the corresponding Scatchard plots (see Figure S2). The values (Fig. 1B) lie in the nanomolar range, which is, however, quite low for SH3-interactions. It is important to emphasize that these results represent apparent Kd-values that hold for the interaction between Sam68 and the SH3-phages, not the isolated SH3 domains (see Discussion). The highest affinity binder is the SH3 domain from the Src-family kinase Yes, followed by Src itself, and Lyn. This is in line with the common notion describing Sam68 as a ligand for Src-family kinases (SFKs) [6]. The newly discovered ligands intersectin 2 #3 and the osteoclast stimulating factor were also confirmed as high-affinity binders. Finally, the analysis confirmed binding of Nck1#2 to Sam68 and ruled out binding of Nck1#1, and Nck1#3. It is unlikely, that the failure of Nck1#1 in binding is due to constraints of the phage system, as the very domain has been selected from the same library as a prime partner for the ligand protein CD3ε [39]. The reason for the discrepancy to the data by Lawe *et al.* [38] with Nck1#1 being the Sam68-binder remains unclear.

To confirm, that the selected SH3 domains are also capable of interacting with endogenous Sam68 from eukaryotic cells, which contains several post-translational modifications [6], we performed GST-pull-down-assays using recombinant GST-SH3-fusion proteins. The latter were immobilized on epoxy-activated magnetic beads and incubated with a whole cell lysate from the human T-cell line MT-4. After thorough washing, bound protein was eluted with SDS-PAGE sample buffer and analysed by Western Blot analysis (Fig. 1C). As expected, all SH3 domains were able to capture Sam68 with varying effectivity, while the negative controls (GST-RasGAP-SH3 and GST only) did not bind Sam68. Overall, the apparent signal intensities correlate with the affinities deduced from the phage-ELISA-analysis, with some variations. For example, GST-Fyn-SH3 retained much more Sam68 than GST-Nck1#2-SH3, despite a higher Kd-value in the ELISA, while Src-SH3 or p85α-SH3 bound less Sam68.

### Characterization of High-affinity Sam68-binders in vivo

**Figure 2:**
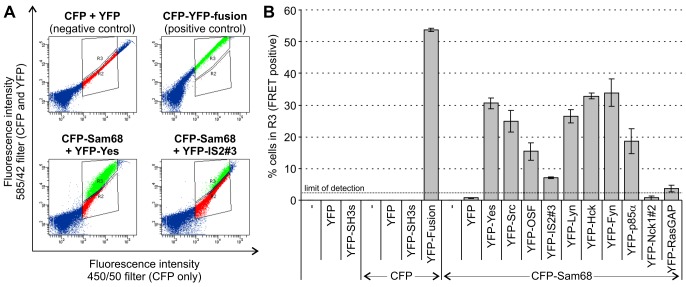
Analysis of Sam68-binding SH3 domains *in vivo* by FRET-analysis. Expression constructs for CFP-tagged Sam68 and YFP-tagged SH3 domains were used to co-transfect 293T cells as indicated. 48 h post transfection, cells were harvested for flow cytometric analysis. Direct protein interaction *in vivo* was assayed by determining FRET from CFP to YFP by exciting CFP at 405 nm and measuring fluorescence with filters 450/50 (CFP only) vs. 585/42 (CFP + YFP-FRET-signal). (A) Representative diagrams showing the shift of cell populations as a result of FRET. Based on the negative control (CFP, or CFP-Sam68, and YFP on separate plasmids) and the positive control (CFP-YFP-fusion protein on one plasmid) two gates were defined, enclosing cells that do not exhibit FRET (R2, red), or that do exhibit FRET (R3, green), which is manifest by a shift to the left (i.e. lower CFP emission) and simultaneously to the top (i.e. higher YFP-emission). The degree of this shift depends on the FRET-efficiency. (B) FRET signals for all domains assayed. Results are shown as mean ± standard deviation from three independent experiments.

To confirm that the SH3 domains can in principal also interact with Sam68 in living cells, we applied a FRET-analysis adapted from [34] making use of CFP-tagged Sam68 and YFP-tagged SH3 domains. Expression constructs for both were used to cotransfect 293T cells, which were analysed 48 h later for CFP and YFP-fluorescence by flow cytometry. In case of a direct interaction, i.e. co-localization at a distance of not more than 10 nm, part of the energy from excited CFP is transferred to YFP, thus increasing YFP emission while simultaneously reducing CFP emission. This can easily be visualized in the FACS-plots with quantification of the magnitude of the effect being possible by defining appropriate gates (see Fig. 2A). The results for all interaction pairs are shown in Fig. 2B. As negative control, coexpression of CFP and YFP on their own yields no FRET-signal, while a CFP-YFP-fusion-protein yields the highest FRET-signal, as expected. As for the interaction of CFP-Sam68 with YFP-SH3 domains, varying degrees of interaction are observed, while overall results correlate quite well with the above results, again exhibiting relative differences in detail. Unexpectedly, a small but significant signal was observed for the interaction with RasGAP.

### Identification of Sam68-PxxP-motifs Engaged in SH3 Domain Binding

Knowledge of the exact binding sites for the vast number of SH3 ligands will be necessary for understanding the complex interplay of Sam68 with its many partners. Limited analyses have been performed for certain SH3 domains, however, the data presented is not complete, as no study has so far comprehensively analyzed binding to all seven motifs. Therefore, we systematically assessed which of the seven PxxP motifs (denoted P0 to P6) serve as binding-sites for SH3 domains and whether differential binding of the various domains takes place. We produced 18–20 aa long peptides comprising the central PxxP motif and its flanking residues (see. Table S1), fused to GST as scaffold for purification. These purified GST-Px-peptides were used as target proteins in a phage-ELISA as described above. The results are summarized in Table 2. Obviously, only the proline-rich motifs P0, P3, P4 and P5 constitute target sites for SH3 domains. Members of the Src-kinase family share a similar binding profile, exhibiting interactions with all four crucial PxxP motifs, except for Fyn, which has overall the lowest affinity to recombinant Sam68 among the SFKs (compare Fig. 1B). Probably, binding of Fyn to P0 and P4 does also occur, but with an affinity below the limit of detection of the phage-ELISA. In all cases, SFKs exhibit highest affinity towards motif P5, followed by P3 and P0, while affinity to P4 is lowest. This picture is remarkably different for the non-SFK SH3 domains tested here, including intersectin 2 and the osteoclast stimulating factor, none of which bind to P5, while P0 seems to be the most important determinant for binding to Sam68.

**Table 2:** Identification of Sam68-PxxP-motifs responsible for SH3-domain-binding.

SH3 domain		P0	P1	P2	P3	P4	P5	P6
Src kinases	Fyn				+		++	
	Hck	++			++	+	++	
	Lyn	+++			+++	++	+++	
	Src	++			++	++	+++	
	Yes	+			++	+	+++	
Others	IS2#3	+						
	Nck1#2	+			+			
	OSF	+			+			
	p85α	++				+		
Negative control	RasGAP							

Peptides corresponding to the seven PxxP-motifs (P0 to P6) of Sam68 were purified as GST-fusions and analyzed for interaction with the indicated SH3 domains by phage-ELISA. Results are expressed semi-quantitatively as half-maximal binding occuring at <10^11^ (+++), 10^11^−10^12^ (++), >10^12^ cfu/ml (+).

Overall, affinities of the SH3 domains to any PxxP motif were lower than to the full-length protein. However, considering that the SH3 domains bind to more than one PxxP-motif as evidenced here, an avidity effect might be in operation (see Discussion).

*Generation of Sam68 mutants with inactive SH3 binding sites -* The motifs P1, P2 and P6 did not show binding to SH3 domains in the above described peptide-analysis, but might be functional in the context of the full-length protein. To exclude this, we analyzed Sam68-mutants with inactivated P0, P3, P4, and P5 for their capacity to bind SH3 domains. In generating these mutants, we aimed at introducing the least possible number of point mutations, since any change, especially of a proline residue, might negatively influence folding and concomitantly other functions of Sam68. Therefore, in the first instance, we designed a panel of PxxP-peptide mutants, containing different point mutations (see Table 3) and checked for alterations in SH3 domain binding. Based on the results shown above, only motifs P0, P3, P4, and P5 were analyzed for those SH3 domains exhibiting the respective binding profiles. The mutant peptides were produced as GST-fusions and analyzed by phage-ELISA like their wildtype counterparts for loss of binding (see Table 3). Motif P0 can be rendered inactive by changing the C-terminal arginine to alanine with the core PxxP remaining untouched, emphasizing the often observed importance of a basic amino acid near the PxxP in many SH3 target sequences. Alternatively, P0 function is reduced by mutating any one of the prolines, and completely lost by mutating both. Mutating prolines in P3 leads to a gradual loss of binding with complete inactivation of motif P3 requiring the replacement of all five intertwined prolines by alanines. Inactivation of motif P4 readily occurs by exchanging the first proline, whereas mutating the N-terminal arginine leads to a reduction, albeit not a complete loss of binding. Motif P5 can formally be broken down into three independent intertwined PxxP motifs, two directly consecutive ones with a third woven into their xx residues (pxPppPxp). Remarkably, analysis of mutants thereof demonstrated that only the central motif constitutes the SH3 binding site, while mutation of the remaining prolines had no impact on ligand binding. Moreover, exchange of the first central proline by alanine is again sufficient to render the motif inactive. Based on these results, Sam68 mutants were designed with any one motif singly inactivated (Sam68ΔP0, Sam68ΔP3, Sam68ΔP4, and Sam68ΔP5), or all motifs inactivated at once (Sam68ΔP0345), introducing the least possible number of mutations.

**Table 3:** Interaction of Sam68 PxxP peptide mutants with SH3 domains.

	P0					P3								P4					P5					
	W	M1	M2	M3	M4	W	M1	M2	M3	M4	M5	M6	M7	W	M1	M2	M3	M4	W	M1	M2	M3	M4	M5
SH3 domain	PxxPxR	PxxPxA	AxxPxR	PxxAxR	AxxAxR	<b>PPxPPxP</b>	<b>APxAPxP</b>	<b>PAxPAxP</b>	<b>PPxAPxA</b>	<b>AAxAPxP</b>	<b>APxAPxA</b>	<b>AAxAPxA</b>	<b>AAxAAxA</b>	RxxPxxP	AxxPxxP	RxxAxxA	RxxAxxP	AxxAxxP	<b>PxPPPPxP</b>	<b>AxPAPPxP</b>	<b>PxPPAPxA</b>	<b>PxAPPAxP</b>	<b>AxAPAPxP</b>	<b>PxAPPPxP</b>
Yes	**<b>++</b>**	**<b>0</b>**	**<b>0</b>**	**<b>0</b>**	**<b>0</b>**	++	+	++	+	0	0	0	0	**<b>+</b>**	**<b>0</b>**	**<b>0</b>**	**<b>0</b>**	**<b>0</b>**	++	++	++	0	0	0
Src	**<b>++</b>**	**<b>0</b>**	**<b>++</b>**	**<b>++</b>**	**<b>0</b>**	++	++	++	++	+	+	+	0	**<b>++</b>**	**<b>+</b>**	**<b>0</b>**	**<b>0</b>**	**<b>0</b>**	++	++	++	0	0	0
Lyn	**<b>++</b>**	**<b>0</b>**	**<b>++</b>**	**<b>++</b>**	**<b>0</b>**	++	++	++	++	++	++	+	0	**<b>++</b>**	**<b>+</b>**	**<b>0</b>**	**<b>0</b>**	**<b>0</b>**	++	++	++	0	0	0
Hck	**<b>++</b>**	**<b>0</b>**	**<b>0</b>**	**<b>0</b>**	**<b>0</b>**	++	++	++	++	+	+	0	0	**<b>n/a</b>**	**<b>n/a</b>**	**<b>n/a</b>**	**<b>n/a</b>**	**<b>n/a</b>**	++	0	0	0	0	0
Fyn	**<b>n/a</b>**	**<b>n/a</b>**	**<b>n/a</b>**	**<b>n/a</b>**	**<b>n/a</b>**	++	0	0	0	0	0	0	0	**<b>n/a</b>**	**<b>n/a</b>**	**<b>n/a</b>**	**<b>n/a</b>**	**<b>n/a</b>**	++	++	++	0	0	0
OSF	**<b>++</b>**	**<b>0</b>**	**<b>0</b>**	**<b>0</b>**	**<b>0</b>**	++	+	+	+	+	0	0	0	**<b>n/a</b>**	**<b>n/a</b>**	**<b>n/a</b>**	**<b>n/a</b>**	**<b>n/a</b>**	n/a	n/a	n/a	n/a	n/a	n/a
IS2#3	**<b>++</b>**	**<b>0</b>**	**<b>0</b>**	**<b>0</b>**	**<b>0</b>**	n/a	n/a	n/a	n/a	n/a	n/a	n/a	n/a	**<b>n/a</b>**	**<b>n/a</b>**	**<b>n/a</b>**	**<b>n/a</b>**	**<b>n/a</b>**	n/a	n/a	n/a	n/a	n/a	n/a
p85α	**<b>++</b>**	**<b>0</b>**	**<b>0</b>**	**<b>0</b>**	**<b>0</b>**	n/a	n/a	n/a	n/a	n/a	n/a	n/a	n/a	**<b>+</b>**	**<b>0</b>**	**<b>0</b>**	**<b>0</b>**	**<b>0</b>**	n/a	n/a	n/a	n/a	n/a	n/a
Optimal	**<B>M1 = </B>** PxxPxA					M7 = **<b>AAxAAxA</b>**								**<B>M3 = </B>** RxxAxxP					M5 = **<b>PxAPPPxP</b>**					

W  =  wildtype, M1 to M7  =  mutants, n/a  =  not applicable.

Compilation of phage-ELISA results indicating changes in binding of the SH3 domains to the PxxP-peptide-mutants compared to the wildtype peptide (++), classified as affinity reduced (+) or interaction abolished (0).

Core residues of the PxxP motifs are underlined; introduction of alanine point mutations is highlighted by a black background.

**Figure 3:**
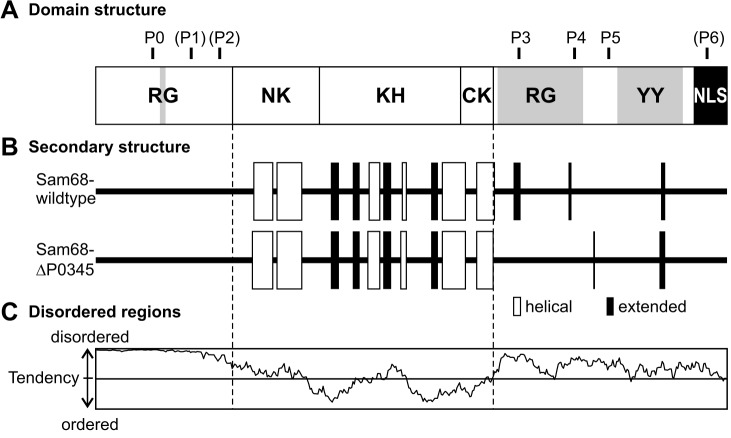
Secondary structure prediction of wildtype and mutant Sam68. (A) Schematic representation of Sam68 domains and positions of proline-rich motifs. The three motifs not binding to SH3 domains are enclosed in brackets. RG  =  arginine glycine rich region, NK  =  N-terminal of KH domain, KH  =  hnRNP K homology domain, CK  =  C-terminal of KH domain, YY  =  tyrosine rich region, NLS  =  nuclear localization sequence (B) Prediction of secondary structure by JPred, white bars: helical regions, black bars: extended regions. (C) Prediction of intrinsically disordered regions by IUpred.

To estimate if the eight point mutations introduced into Sam68 (seven of them affecting proline) negatively influence its structure, we performed secondary structure prediction using JPred [40]. As shown in Fig. 3A, the regions ecompassing the four PxxP motifs locate outside the central GSG domain, which almost exclusively harbors secondary structural elements (Fig. 3B). The algorithm only predicts very short stretches of extended protein backbone conformations in the C-terminal part of Sam68 which most likely do not contribute to an overall 3-D fold. This finding is in line with a prediction of intrinsically disordered regions (Fig. 3C) performed with IUPred [41] that shows a very high disorder tendency for the entire region N-terminal of the GSG domain, as well as for most of the region C-terminal of the GSG domain. These *in silico* data implicate a structural model of Sam68 that comprises a well-folded central domain for RNA binding flanked by unstructured tails that serve as docking sites for diverse interaction partners. This theme is not uncommon, as intrinsically disordered regions offer greater flexibility for multiple interactions with signalling proteins [42]. Performing the predictions again for the Sam68ΔP0345 mutant indicates, that, as anticipated, folding of the central GSG domain is not impaired. Thus, we expect no alterations in Sam68 structure and function, except for the desired impairment of binding to SH3 domains.

**Figure 4:**
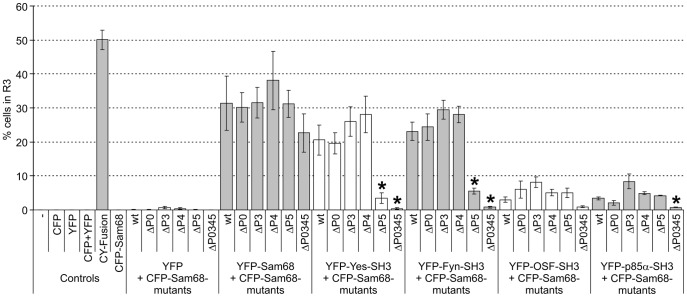
Interaction of Sam68-PxxP-mutants with SH3 domains. Expression constructs for CFP-tagged Sam68-mutants defective in either any one of the SH3-interacting PxxP-motifs (Sam68ΔP0, −ΔP3, −ΔP4, −ΔP5), or defective in all (Sam68ΔP0345), were cotransfected with YFP-tagged SH3 domains from Yes, Fyn, p85α, or OSF, or wildtype-Sam68 into 293T-cells and analyzed for direct interaction *in vivo* by performing FRET-analysis as in Fig. 2. Results are shown as mean ± standard deviation from three independent experiments. Significant reduction (p<0.05 in Student’s T-test) of the signal as compared to wildtype is marked with an asterisk.

To verify the modulation of the SH3 binding capacity in a cellular context, interaction of the Sam68ΔPxxP mutants with SH3 domains was assessed by FRET-analysis using CFP-Sam68ΔPxxP-constructs and the described YFP-SH3s. Fig. 4 shows the results for SH3 domains from two members of the SFKs (Yes and Fyn) and two other Sam68-binders (OSF and p85α). Direct detection of CFP-Sam68-ΔPxxP mutant expression via measurement of the CFP fluorescence showed that all mutants are produced at similar levels. Furthermore, the GSG-domain-dependent self-association with YFP-tagged wildtype-Sam68 was similar for all variants tested. This affirms the assumption that introduction of the point-mutations did not cause a general protein-defect. FRET-analysis of the Sam68-mutants’ interactions with Yes and Fyn yields similar results in agreement with the *in vitro* data from the phage-ELISA showing similar binding profiles for all SFKs. Single inactivation of motifs P0, P3 and P4 does not eliminate the interaction of SH3 domains with Sam68, due to the remaining intact motifs still mediating the interaction. Inactivation of P5, however, causes a significant decrease of the FRET-signal, confirming the observation that P5 is the highest affinity motif. Signal reduction to the background-level is not observed until all four motifs are disrupted in combination. OSF-SH3 exhibited similar binding to P0 and P3 in the ELISA-analysis, which is recapitulated in the FRET assay. Only for the Sam68ΔP0345 mutant, binding to OSF-SH3 is impaired. The same is true for the SH3 domain of p85α, though a slight but non-significant tendency of reduction is visible for Sam68ΔP0. In conclusion, by introducing eight rationally defined point-mutations affecting the four relevant PxxP motifs, the Sam68ΔP0345 mutant, being incapable of binding to SH3 domains any more, could readily be generated. This eventually confirms the absence of SH3-binding functionality of the remaining intact proline-rich motifs P1, P2, and P6.

## Discussion

The protein Sam68 is a well-known SH3-domain binder comprising an exceptionally large number of seven potential PxxP ligand motifs. To comprehensively characterize the SH3 binding potential in an unbiased manner, we conducted a phage-display-based screening of Sam68 against a library containing the entire human SH3 proteome. Thereby we identified twelve high-confidence binders, five of which are described for the first time to our knowledge. Furthermore, we identified a set of 48 SH3 domains, which might contain lower-affinity interactors, among them again some already known Sam68-binders such as Grb-2 or Vav1. Extension of the analysis would presumably have led to the classification of more domains as high-confidence binders, and to the identification of more lower-affinity binders, as even some of the already known binders remain undetected. Moreover, in the case of proteins with more than one SH3 domain, cooperative binding to different PxxP motifs might be necessary for a high-affinity interaction [43]. As these domains are presented separately on different phages, such proteins might elude identification in the bio-panning, thus possibly explaining why e.g. Grb-2, which has been shown to bind to Sam68 via both of its SH3 domains [44], was only among the lower-affinity binders. Finally, we cannot rule out that the structure of individual SH3 domains is compromised on the phage surface.

For an SH3 domain subset consisting of the highest-affinity binders, we confirmed the Sam68-interactions in independent assays, i.e. *in vitro* by GST-SH3-pull-down-assays and *in vivo* by FRET-analysis using fluorescent-protein-fusions. However, differences in the relative interaction strengths were observed between the various assays for some pairs (compare e.g. affinity of Src in the ELISA with the band intensity in the pull-down assay, or Fyn in the ELISA vs. the FRET-analysis). Most likely these differences are due to post-translational modifications of Sam68, which influence its interaction capacities. As mentioned in the introduction, Sam68 is subject to S/T-phosphorylation [30], Y-phosphorylation [45], acetylation [15], methylation [14], or sumoylation [22]. Furthermore, the affinites may be influenced by assay-specific constraints. For instance, the FRET efficiency also depends on the spatial orientation of both fluorophors towards each other, which might vary for the different SH3-YFP fusion proteins despite very high similarity in the overall structure.

Our analysis of the very PxxP-motifs engaging SH3 domains shows a delicate selectivity of certain motifs and, considering the three intertwined but formally separable PxxPs of P5, or the basic aa in P0 and P4, even the importance of individual amino acids. Only P0, P3, P4, and P5 constitute SH3-domain target sites. Mutations in Sam68 inactivating these four motifs suppressed any interactions with SH3 domains, thus ruling out functionality of P1, P2 and P6 as SH3 ligands. The absence of SH3-interactions of P1, P2, and P6 suggests SH3-independent functions of these motifs, i.e. interactions with other domains recognizing proline-rich sequences, like WW-domains [46], [47]. The various SH3 domains have special preferences to the four motifs concerning selectivity and affinity. The recognition pattern of Src-kinase-family SH3 domains is quite similar, with major preference for P5, while it is completely different to the pattern of e. g. intersectin 2 or the osteoclast stimulating factor.

In conclusion, the diverse preferences of the different SH3 domains for certain PxxP-motifs constitutes a prime example for the high selectivity of SH3 domains for their target sequences. Moreover, in the cellular context, it is conceivable that yet an increase in specificity is achieved for proteins with more than one SH3 domain (i.e. Intersectin 2 (5 SH3s), Nck1 (3 SH3s), CIN85 (3 SH3s)) by cooperative binding to different PxxP motifs of Sam68, as it has been suggested for the interaction of Nck1 with its binding partner Cbl [43], [48].

To rank SH3 domain affinities towards Sam68 a phage-ELISA analysis was performed. As outlined in the results section, calculation of Kd-values relies on the estimation of the mean number of SH3-domains present on one phage particle. This number was deduced from a comparison with the Nef-Hck-SH3 pair, for which a Kd-value of 250 nM has been determined by surface plasmon resonance measurements [5]. Thereby, we obtained a value of 420 pVIII-SH3 proteins (SH3 domain ≈7 kDa), corresponding to 26 % of the approx. 1600 pVIII surface proteins. This number is plausible when compared to values from the literature: Short 15-meric peptides (≈1.7 kDa) are incorporated as pVIII-fusions at 30–40 % [49], while antibody-Fab-fragments (≈50 kDa) are only incorporated at less than 1% [50].

Remarkably, Kd values calculated for the Sam68-SH3-interactions (considering the aforementioned correction value) lie in the low nanomolar range (cf. Fig. 1B). This is unexpected for SH3 domains, whose affinities are considered to lie in the low micromolar range [2], [51]. However, critical examination of the literature challenges the generality of the latter proposition. Several examples can be found for much better SH3-interactions (e.g. Pak2 with β-Pix-SH3 at 59 nM [31]), and Kd values for SH3-domains have often been determined only for short peptide-ligands and not the whole proteins. This can have a significant influence on binding-strength, as illustrated for instance for the Abp1-SH3 domain, comprising a Kd-value of 100 µM to a 14-mer ligand-peptide, and 40 µM after elongation to a 17-mer peptide [52]. Nevertheless, some values obtained for Sam68-SH3 interactions still are one order of magnitude lower than even the best reported in the literature. Likely, this is due to an artifical avidity effect resulting from the use of the SH3-phages. As the SH3 domains bind to more than one of the PxxP-motifs, it is conceivable that one phage-particle docks to two or more PxxP-motifs of an individual Sam68 molecule via multiple SH3 domains. Consequently, even after dissociation of one SH3-PxxP-pair, the phage would still be retained by the protein. Kinetically, this corresponds to a decrease in the off-rate and concomitantly to a decrease in the Kd value. The affinity gain of the interaction is not due to cooperativity, as is evident from the Hill-transformed ELISA data yielding Hill-coefficients α of 1.0. Rather, the increase can simply be attributed to enhancement as defined by Mammen *et al.* [53] due to the polyvalent nature of the interaction. In fact, binding curves from phage-ELISAs with the PxxP-peptides instead of full-length Sam68 indicate weaker interactions, supporting the above observation of binding enhancement. In conclusion, the given data represent the apparent Kd-values of the interaction between SH3-phages and Sam68, which nevertheless allow for comparison of SH3-domain binding stengths on a relative scale.

Apart from those SH3 domains binding Sam68 with high affinity that have already been described in the literature, we identified five new ones: Intersectin 2 (IS2), nephrocystin, sorting nexin 9, Cbl-interacting protein of 85 kDa (CIN85), and Osteoclast stimulating factor 1 (OSF).

Intersectins 1 and 2 are implicated in Clathrin-dependent endocytosis [54]. They comprise a number of protein-interaction domains, among others five SH3 domains each. Intersectins are considered as scaffold-proteins organizing components of the endocytosis machinery. A similar function is ascribed to the Cbl-interacting protein CIN85, which facilitates endocytosis of receptor tyrosine kinases after activation by ligands [55]. Sortin nexin 9 is involved in endocytosis as well, likely by linking the key GTPase dynamin to the actin cytoskeleton [56]. Notably, some Sam68-binding SFKs are implicated in endocytotic processes as well, like Hck, which is involved in the regulation of actin-dependent processes during phagocytosis [57]. Taken together, the identification of several Sam68-binders that are involved in endocytosis strongly suggests a so far unknown function of Sam68 in this central biological process. Endocytosis plays an important role in many signalling processes such as activation of the MAP-kinase cascade [58], and Sam68 might be engaged in cross-talk of these processes.

As implicit in the name, the osteoclast stimulating factor (OSF) plays an important role in osteoclast differentiation. It has been shown that expression of *osf* leads to secretion of a so-far unknown factor, which induces differentiation of hematopoietic stem cells into osteclasts in cell culture [59]. Furthermore, OSF interacts with Src, which is a noteworthy connection, as Src^-/-^ knock-out mice exhibit major bone deformations due to impaired osteoclast function leading to osteopetrosis [60]. Integrating the observation that the Sam68^−/−^ knock-out mouse exhibits an osteopetrosis-phenotype as well [28], and the interaction between Sam68 and OSF, suggests a picture of an osteoclast-specific signal transduction pathway containing Src, OSF, and Sam68. The latter possibly facilitates phosphorylation of OSF by Src, functioning as a platform that brings both proteins close together. This view might help to understand the osteopetrosis phenotype of the Sam68^−/−^ knock-out mouse on a molecular level. Interaction of OSF with Src is in principle still possible, but maybe only occurs inefficiently, presumably translating into the milder bone-related phenotype for knock-out of Sam68 than for Src.

Similar roles in facilitating certain steps of signal transduction pathways are often carried out by scaffold proteins, a heterogeneous group of unrelated proteins. Classical scaffold proteins are defined by three criteria according to Zeke *et al.* [61]: (i) They possess no signalling-related catalytic activity by themselves, but (ii) directly interact with at least two proteins of a signalling pathway, that (iii) form a pair of a catalytically active protein and its corresponding target. Sam68 lacks catalytic activity and binds to a multitude of proteins even when putting the numerous SH3 domains aside, thus complying with the first two criteria. Regarding the third criterion, the here described OSF-Src-interaction is satisfactory. In principal, this characteristic has already been recognized by Richard *et al.* for a different protein-pair, namely an SFK-member and phospholipase C gamma 1 (PLCG1). In their proposed model, the SFK phosphorylates PLCG1 after both proteins made contact to Sam68 [37]. Thus, our findings support their original farsighted proposition, and together the findings suffice to formally consider Sam68 as a *bona fide* classical scaffold protein. However, Sam68 is unique in this group in two regards: First, it is predominantly located in the nucleus rather than in the cytoplasm like common scaffold proteins, and second, it is capable of binding RNA, thus adding another degree of complexity to the scaffolding-property. Hopefully, this view will help to better understand the multiple roles that Sam68 plays in the many different biological processes it is involved in. This demands the identification and characterization of the relevant Sam68-ligands, which actually mediate a certain function that is facilitated by Sam68.

## Supporting Information

Figure S1 **Phage-ELISA analysis: Recombinant His-Nef (green line) or His-Sam68 (blue line) were coated in 96-well-plates and incubated with dilution series of individual SH3-phage supernatants.** The amounts of bound phages were measured using an HRP-conjugated anti-M13-specific antibody with TMB-substrate detection at 450 nm. Data shown is the mean of three independent experiments (normalized to maximum OD-values) ± standard deviation of OD values (vertical) and phage titration (horizontal).
(TIF)

Figure S2 **Depiction of data from** **Fig. 1A** **in the main text with values transformed as Scatchard Plot. The slopes of the regression lines correspond to −1/Kd.**
(TIF)

Table S1 **PxxP motifs of Sam68.**
(DOC)

Table S2 **SH3 domains binding to Sam68 according to results published in the literature (the data in the table was compiled to the best of our knowledge).**
(DOC)

Table S3 **Sam68-binding SH3 domains as identified by bio-panning of His-tagged or GST-tagged Sam68 against the human SH3-proteome phage-display library.**
(DOC)
