# Supplementary material for: Comprehensive Analysis of Interactions between the Src-Associated Protein in Mitosis of 68 kDa and the Human Src-Homology 3 Proteome
Source: PLoS One. 2012 Jun 20;7(6):e38540. doi: 10.1371/journal.pone.0038540 (PMC3379994; doi:10.1371/journal.pone.0038540)
Supplement: Table S1 — PxxP motifs of Sam68. (DOC) [file pone.0038540.s003.doc]

**Supplementary Table S1**

| Name | Sequence | Type ^a^ | Position |
| --- | --- | --- | --- |
| P0 | ...QTPSRQ**P**PL**P**H**R**SRGGGG... | Class 2R | 32 - 50 |
| P1 | ...ASPATQPP**P**LL**P**PSATGPD... | PxxP-Consensus | 57 - 75 |
| P2 | ...ATVGGPAPT**P**LL**P**PSATASV... | PxxP-Consensus | 76 - 95 |
| P3 | ...RGRGAA**PP**P**PP**V**P**RGRGVG... | PxxPxxP and  PxxP-Consensus | 289 - 305 |
| P4 | ...RGATVT**R**GV**P**PP**P**TVRGAP... | Class 1R | 325 - 343 |
| P5 | ...AGIQRI**P**L**PPPP**A**P**ETYEEY... | PxxPPxxP and  PxxP-Consensus | 350 - 369 |
| P6 | ...GTRPSL**K**AP**P**AR**P**VKGAYR... | Class 1K | 418 - 436 |

^a^ Classification of PxxP motifs according to [3]
