# Supplementary material for: Comprehensive Analysis of Interactions between the Src-Associated Protein in Mitosis of 68 kDa and the Human Src-Homology 3 Proteome
Source: PLoS One. 2012 Jun 20;7(6):e38540. doi: 10.1371/journal.pone.0038540 (PMC3379994; doi:10.1371/journal.pone.0038540)
Supplement: Table S2 — SH3 domains binding to Sam68 according to results published in the literature (the data in the table was compiled to the best of our knowledge). (DOC) [file pone.0038540.s004.doc]

**Supplementary Table S2**

| Protein | | References |
| --- | --- | --- |
| Src kinases | Brk/Sik | [62], [63] |
|  | Fgr | [64] |
|  | Fyn | [65], [11], [66], [37], [47], [67], [35], [68] |
|  | Hck | [69] |
|  | Lck | [67], [69], [70], [71] |
|  | Lyn | [66] |
|  | Src | [64], [65], [66], [67], [35], [21], [72] |
|  | Yes | [35], [69] |
| Others | Btk | [73] |
|  | Crk | [69], [21] |
|  | Grap | [74] |
|  | Grb2 | [67], [68], [69], [72], [74], [44] |
|  | Itk | [67], [69], [75] |
|  | Nck1 #1 | [38] |
|  | p47phox | [64] |
|  | PI3K-p85α | [64], [35], [21], [72] |
|  | PLCγ1 | [64], [37], [47], [35], [72] |
|  | PRMT2 | [35] |
|  | Tec | [69] |
|  | Vav | [76] |
