# Supplementary material for: Comprehensive Analysis of Interactions between the Src-Associated Protein in Mitosis of 68 kDa and the Human Src-Homology 3 Proteome
Source: PLoS One. 2012 Jun 20;7(6):e38540. doi: 10.1371/journal.pone.0038540 (PMC3379994; doi:10.1371/journal.pone.0038540)
Supplement: Table S3 — Sam68-binding SH3 domains as identified by bio-panning of His-tagged or GST-tagged Sam68 against the human SH3-proteome phage-display library. (DOC) [file pone.0038540.s005.doc]

**Supplementary Table S**3

| **Nr.** | **SH3 domain** | **Accession Number** | **Frequency**  GST-Sam68  (out of 78) | **Frequency**  His-Sam68  (out of 84) | **Frequency**  combined  (out of 162) | **Known** |
| --- | --- | --- | --- | --- | --- | --- |
| 4 | AblBP3 / Abi-2 | Q13249 | 0 | 1 | 1 |  |
| 7 | AHI-1 | Q9H803 | 1 | 0 | 1 |  |
| 9 | Amphiphysin | P49418 | 1 | 2 | 3 |  |
| 19 | ARHGEF9 | NP_056000 | 0 | 2 | 2 |  |
| 20 | Asef / ARHGEF4 | Q9HDC6 | 1 | 0 | 1 |  |
| 21 | ASPP1 | cac83011 | 0 | 1 | 1 |  |
| 23 | IRTKS | Q9UHR4 | 1 | 0 | 1 |  |
| 28 | Btk | Q06187 | 0 | 1 | 1 | + |
| 36 | CD2BP1 | AAH08602 | 0 | 1 | 1 |  |
| 37 | CIN85 #1 | Q9NYR0 | 2 | 2 | 4 |  |
| 40 | CMS #1 | Q9Y5K6 | 1 | 2 | 3 |  |
| 42 | CMS #3 | Q9Y5K6 | 1 | 0 | 1 |  |
| 52 | DLG1 | Q12959 | 1 | 0 | 1 |  |
| 53 | DLG2 | Q15700 | 1 | 1 | 2 |  |
| 54 | DLG3 | Q92796 | 1 | 1 | 2 |  |
| 55 | DLG4 / PSD95 | P78352 | 1 | 0 | 1 |  |
| 58 | DOCK2 | Q92608 | 1 | 0 | 1 |  |
| 59 | DOCK3 | AAN12301 | 0 | 1 | 1 |  |
| 83 | Fgr | P09769 | 0 | 1 | 1 | + |
| 84 | FISH #1 | Q9H462 | 1 | 0 | 1 |  |
| 93 | Frk/Rak | P42685 | 1 | 0 | 1 |  |
| 95 | Fyn | P06241 | 9 | 2 | 11 | + |
| 104 | Grb2 #1 | P29354 | 0 | 1 | 1 | + |
| 105 | Grb2 #2 | P29354 | 1 | 0 | 1 | + |
| 106 | Hck | P08631 | 2 | 5 | 7 | + |
| 112 | Homology to PRAX-1 #2 | Q9UFD9 | 2 | 1 | 3 |  |
| 116 | Intersectin 1 #3 | O95216 | 0 | 2 | 2 |  |
| 120 | Intersectin 2 #1 | Q9NYG0 | 1 | 0 | 1 |  |
| 122 | Intersectin 2 #3 | O95062 | 2 | 6 | 8 |  |
| 125 | Itk | Q08881 | 0 | 1 | 1 | + |
| 132 | Lyn | P07948 | 8 | 8 | 16 | + |
| 135 | MIA2 | aal26990 | 0 | 1 | 1 |  |
| 146 | MPP6 / VAM1 | Q9H0E1 | 0 | 1 | 1 |  |
| 148 | MYOSIN I-C | Q12965 | 1 | 0 | 1 |  |
| 150 | Myosin VII-a | Q13402 | 1 | 0 | 1 |  |
| 155 | Nck1 #2 | P16333 | 2 | 3 | 5 | + |
| 156 | Nck1 #3 | P16333 | 1 | 0 | 1 | + |
| 158 | Nck2 #2 | O43639 | 0 | 1 | 1 |  |
| 162 | Nephrocystin | O14837 | 6 | 0 | 6 |  |
| 170 | Osteoclast stimulating factor1 | Q92882 | 2 | 3 | 5 |  |
| 177 | p47-phox #2 | P14598 | 0 | 1 | 1 | + |
| 182 | p85α | P27986 | 3 | 8 | 11 | + |
| 184 | PACSIN1 | Q9BY11 | 1 | 0 | 1 |  |
| 193 | Ponsin #2 | AAK57479 | 2 | 0 | 2 |  |
| 207 | PRAX-1 #3 | O75111 | 1 | 0 | 1 |  |
| 214 | RelA associated inhibitor | Q9Y290 | 0 | 1 | 1 |  |
| 218 | RUSC2 | O15080 | 0 | 1 | 1 |  |
| 221 | SASH1 | O94885 | 0 | 1 | 1 |  |
| 229 | SH3YL1 | AAH08375 | 1 | 1 | 2 |  |
| 242 | SKAP55R | O43349 | 1 | 0 | 1 |  |
| 248 | Sorting nexin 30 | AAH18775 | 0 | 1 | 1 |  |
| 249 | Sorting nexin 9 | Q9Y5X1 | 3 | 2 | 5 |  |
| 250 | SPATA13 | bab71009 | 1 | 0 | 1 |  |
| 251 | SPIN90/VIP54 | Q9NZQ3 | 1 | 0 | 1 |  |
| 252 | Src | P12931 | 1 | 6 | 7 | + |
| 266 | STS1 | AAH07541 | 0 | 1 | 1 |  |
| 276 | Tuba #2 | XP_050742 | 0 | 1 | 1 |  |
| 279 | Tuba #5 | XP_050742 | 1 | 0 | 1 |  |
| 284 | Vav1 #2 | P15498 | 2 | 1 | 3 | + |
| 292 | Yes | P07947 | 6 | 7 | 13 | + |
| 293 | ZDHHC6 | AAH07213 | 0 | 1 | 1 |  |
| 294 | ZO-1 | Q07157 | 1 | 0 | 1 |  |
